# Regression based hybrid machine learning model performance evaluation on air quality index prediction in Kolkata

**DOI:** 10.1038/s41598-026-54918-x

**Published:** 2026-06-10

**Authors:** Muni Lakshmi G K, Mokesh Rayalu G

**Affiliations:** 1 Research scholar, Department of Mathematics, School of Advanced Sciences, , Vellore Institute of Technology, Vellore, India; 2 Associate Professor, Department of Mathematics, School of Advanced Sciences, Vellore Institute of Technology, Vellore, India

**Keywords:** Kolkata, Air quality index, PM2.5, Machine learning, Hybrid models, Climate sciences, Environmental sciences, Environmental social sciences

## Abstract

Air pollution, especially elevated particulate matter concentrations, presents a substantial risk to public health and environmental sustainability in urban regions. By employing machine learning and hybrid ensemble models, this study develops a robust frame work for predicting the Air Quality Index (AQI). A multi-step imputation method was used to preprocess the dataset containing metrological variables and air contaminants in order to handle missing values. AQI was selected as the target variable. To assess the possibility of target dependency, two feature configurations were taken in to consideration: a full-featured set and a reduced set that excluded PM2.5 and PM10. Multiple models were used, including Linear Regression, Decision Tree, Random Forest, Gradient Boosting, KNN, MLP and LSTM as well as ensemble methods like Voting and Stacking regressor. Baseline models, namely persistence and SMA were incorporated for comparative analysis. To assess the performance RMSE, MAE, MAPE, RMSLE and R^2^ with a temporal train test split were used. The Voting regressor achieves the lowest RMSE (10.938) and highest R^2^ (0.974), while the Stacking regressor offers the lowest MAE and MAPE demonstrating the superior performance of ensemble models. The LSTM model captures temporal patterns but performs below ensemble models. Models with fewer features perform noticeably worse, underscoring the significance of particulate matter. SHAP analysis shows PM2.5 and PM10 as the most influential features while robustness analysis supports stable performance.

**Supplementary Information:**

The online version contains supplementary material available at 10.1038/s41598-026-54918-x.

## Introduction

The need to tackle issues of resource misuse, pollution-related public health risks, and environmental degradation have been brought to fore by sustainable development frameworks. The Sustainable Development Goals (SDGs) of the UN have it that the human well-being in the long term should be sustainably based on the balancing of land use, environmental resilience, and prioritizing social policies first^1,2^. Unchecked city development driven by in-migration, housing demand and development projects such as flyovers and metro lines in Kolkata has contributed to increased environmental degradation and loss of forest cover. These problems are aggravated by the lack of a detailed master plan, as green spaces only cover 2% of the total area of the city, which is far less than the recommended 10% area in relation to sustainable urban ecosystems^22^. Excessive contact with motor vehicle emissions and fine particulate contaminants present a critical work health hazard to traffic police officers, who spend much time on busy urban streets. The study is a combination of 5 years of on-ground measurements of the environment and the intensive statistical methods used in analyzing the temporal and seasonal variations of the air quality in Kolkata. Making people aware of the exposure to pollutants and the possible health consequences, the study will contribute to the critical importance of data-driven policies and preventive strategies to protect the health of the urban traffic police officers^23^. These perspectives put AQI studies in India, where the problem of pollution has aggravated as a result of high rates of urbanization and population growth, in a global perspective. The changes in space and time of air pollutants and its meteorological predictors are emphasized in different studies. Longitudinal research in Kolkata has shown that there existed consistent variations in seasons and the impact of festivals i.e. Kali Puja and Diwali as a result of firecracker use^4^. A comparative analysis of Kolkata and Siliguri revealed that the PM2.5 and PM10 concentrations peaked during the monsoon, and pollutants were positively correlated with temperature, thus highlighting the role of the meteorological conditions^5^. The COVID-19 pandemic provided a distinctive “natural experiment,” where in lockdown measures led to significant decreases in key pollutants PM10 (72%), PM2.5 (73%), NO2 (84%), SO₂ (48%), and CO (61%) in Kolkata, thereby demonstrating the substantial impact of anthropogenic activities on air quality^6^. A combination of these studies highlights the fact that the anthropogenic aspects of the issue and the meteorological conditions are central to the changes in the Air Quality Index (AQI). One of the areas that have received considerable literature on research is the predictive capabilities of machine learning (ML) and hybrid models in the prediction of air quality index (AQI). Optimization methods such as the Grey Wolf Optimization-Decision Tree (GWO-DT) have been able to achieve a high level of more than 94% accuracy in Kolkata^7^. In the meantime, ARIMA and Random Forest have proven to be 99% accurate in predicting the pollutants in Indian cities^8^. Several ensemble-based approaches such as sparse spectrum Gaussian process regression and stacked regressors frequently outperform single models on a routine basis^9,15^. Based on the comparison of models, such as SVR, Random Forest, KNN, XGBoost, and Gaussian NB, it has been found out that ensemble and hybrid models are better suited to overcome non-linear and temporal challenges compared to the traditional ones^10–12^.

## Literature review

The 17 Sustainable Development Goals (SDGs) have emerged to be one of the international priorities of the United Nations owing to the increasing population pressures, the unsustainable use of the resources, and the environmental pressures. To protect the environment and human health, it is necessary to find a balance between the use of land and population as well as solve the challenges such as inequality, development, and education^1,3^. Such issues are acute in India in particular due to the rapid urbanization of the nation and especially in the largest cities like Kolkata. The continuous growth of the city has led to increased extent of the built-up areas and reduction in the vegetation cover. This is mostly because of migration^16^. Such transformations exacerbate air quality issues hence we require effective methods of monitoring and predicting them. Over the last few years, machine learning (ML) has been used more to predict the Air Quality Index (AQI). Support Vector Regression (SVR), Random Forest and Cat Boost are some of the most popular models. Random Forest can be the most dependable in those cases, when the issues of imbalance of data are addressed with the assistance of such technique as Synthetic Minority Oversampling Technique (SMOTE)^17^. The experiments in West Bengal indicate that the AQI varies throughout the year with the highest indexes recorded in such places as Kolkata, Howrah, and Bardhhaman during the winter. There are strong negative relationships between AQI and weather parameters between AQI and temperature, relative humidity and precipitation. This shows the importance of weather conditions in adjustments of air quality^18^. Many researchers have performed their studies employing machine learning methods to predict air quality in different locations on Earth. Results of^19^ revealed that XGBoost is the most helpful machine learning model regarding the prediction of the hourly air quality index in Azamgarh. Using their data they concluded that the most significant variables were PM 2.5, PM10, NO2 and SO2. They adopted algorithms such as the Random Forest, SVM, ANN and KNN in^8^ to make forecasts of AQI in 196 cities in India. Random forest performance was 99.79 and the other models had a varying degree of performance. Similarly^20^ found that SVR was better at predicting the air quality index (AQI) in Beijing as compared to other models. They obtained an R 2 value of 0.9776 that confirmed its capability of being used in regression to predict air pollution. Data of the city of Delhi was used to evaluate multiple models by^15,21^ both of whom were working in the context of India. Random Forest algorithm was again and again the most effective one on bigger datasets whereas Decision Tree algorithm scored perfectly on smaller datasets.

This evidence shows that tree-based models are reliable in practice. By applying stacked ensemble frameworks with hierarchical encoding and residual learning gives PM10 predictions more accurate and also stops the loss of temporal data^25^^26^. proposed that by using explainability methods like SHAPE and LIME, physical-informed neural networks with humidity constraints will strengthen the forecasting accuracy and assure physically consistent predictions. By integrating tree-based and neural network approaches, hybrid stacked models have the potential to capture complicated non-linear patterns, which leads to enhanced PM10 forecasting accuracy over traditional and standalone models^27^. Accurate and timely global forecasting can be obtained by using transformer-based frame works which explicitly include temporal validation and country embeddings in order to capture long-term dependencies in PM2.5 risk^28^. The hybrid polynomial regression paired with fully connected neural network is found to the best model with improved results, outperforming other machine learning and deep learning models^29^. The hybrid polynomial regression paired with fully connected neural network is found to the best model with improved results, outperforming other machine learning and deep learning models^30^.

A novel random imputation was utilized to fill missing values by taking samples from feature distribution after interquartile range-based outlier removal. This method is highly useful for data missing completely at random and improves forecasting accuracy^31^. To capture spatiotemporal patterns in PM2.5 data, hybrid BiGRU-1DCNN model was proposed to learn about both temporal dependencies and spatial correlations, which results in higher forecasting accuracy compared to standalone models^32^^33^. proposed that Quantum-inspired temporal convolutional networks (QTCN) has outperformed over QCNN in terms of RMSE, MAPE and R^2^. By employing spatiotemporal correlation analysis in Hybrid models has shown significant improvement across error measures by exhibiting greater efficiency over traditional models^34,37,38^. In PM2.5 forecasting, Hybrid models in combination with WaveNet, 1DCNN, BiLSTM and XGBoost has performed well compared to deep learning models in terms of RMSE, MAE and MSE^35,39^. Complicated spatio temporal patterns were captured in PM2.5 forecasting by using the technique decompose-recompose in hybrid model which combines with 1DCNN-BiGRU^36^.

### Limitations of existing works and contribution of our study

Majority of current research on air quality and PM forecasting centres on distinct models or limited comparison which did not manage to reveal the best model performance in different urban settings. Limited set of evaluation metrics, namely RMSE and R^2^ were used for evaluation and comparison of models. Furthermore, MAPE and RMSLE are also evaluation metrics through which we can estimate the comprehended percentage error and also how stable a prediction is. Extensive use of linear and simple regression models, which often fall short to capture the intricate and nonlinear relationships between climatic factors and pollutant concentrations in some studies. Another limitation is that hybrid ensemble models, such as Voting and Stacking, have not been extensively investigated in numerous city-level PM2.5 forecasting studies, despite their potential for improved generalization.

This paper offers a comprehensive comparative analysis of six machine learning regression models and two hybrid ensemble frameworks to predict PM concentration in Kolkata. The use of traditional models and hybrid ensemble techniques optimize how models’ integration improves performance. For fair and accurate performance of the model standard measures like RMSE, MAE, MAPE, R^2^ and RMSLE were implemented. for complex nonlinear pollutant dynamics within urban atmospheric environments these findings validate the superiority of ensemble modelling.

**Fig. 1:**
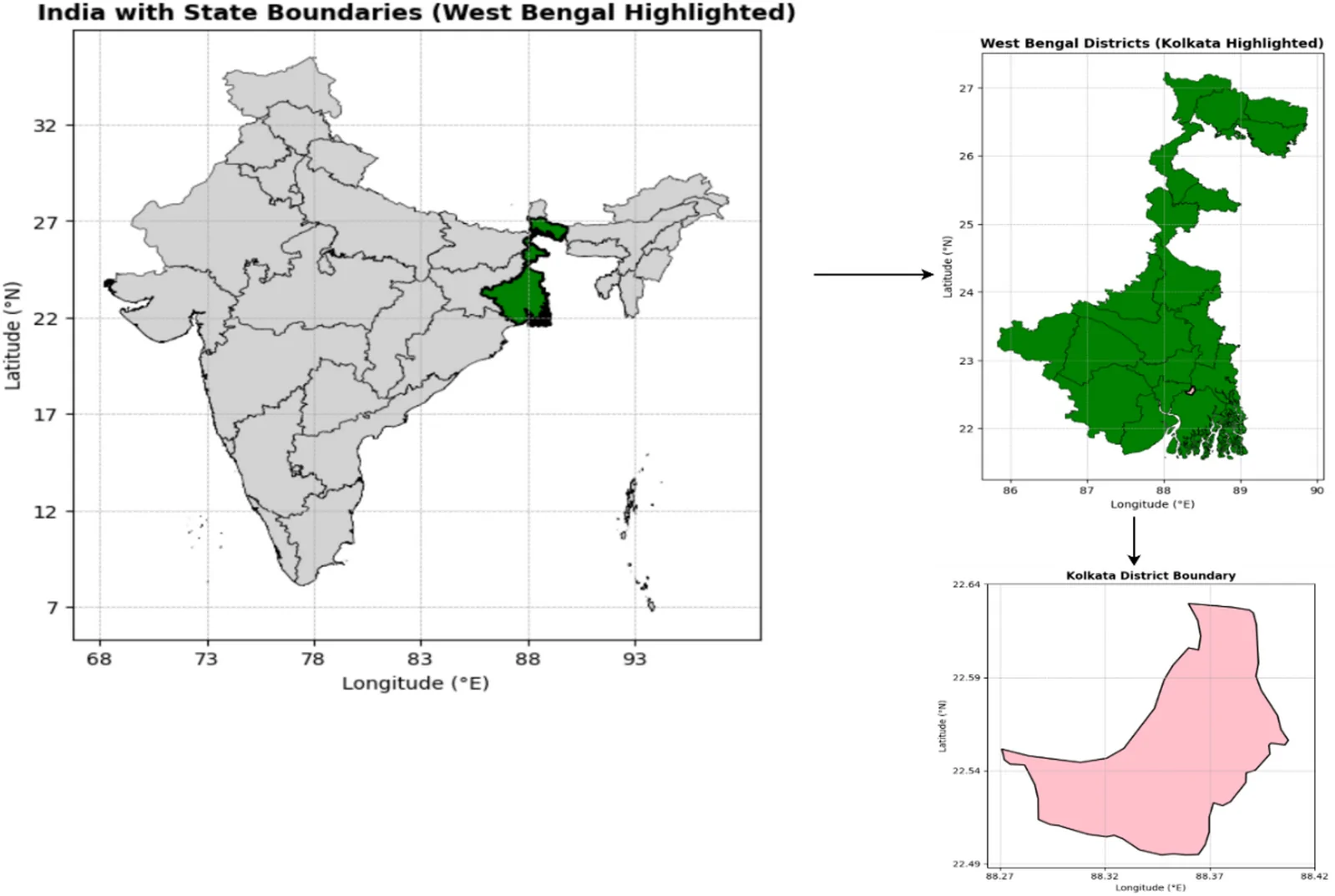
Geographical location of the study area showing the position of Kolkata district with in West Bengal and India, used for AQI prediction and air quality analysis.

## Methodology

### Data description and missing value imputation

#### Data description

The dataset comprises 17,544 hourly observations obtained from CPCB for the city kolkata, India and the study area geographical location is shown in Fig. 1. The study period is from January 2023 to December 2024. It encompasses essential air contaminants and climatic variables affecting air quality. The Air Quality Index (AQI) functions as the dependent variable, whereas all other variables act as independent characteristics. The data is indexed by hour for time-series analysis.

**Table 1:** Input variables, their categories, and units used for AQI prediction.

Category	Variables with units	Description
Pollutant	PM2.5, PM10, NO, NO_2_, NO_X_, SO_2_, O_3_, Benzene, Toluene, Eth-Benzene, MP-Xylene (µg/m³)	Major air pollutants including particulate matter, Nitrogen oxides and volatile organic components (VOCs)
Meteorological	AT (°C), RH (%), WS (m/s), WD (°), RF (mm), TOT-RF (mm), SR (W/m²), BP(hPa), VWS (m/s)	Weather related parameters influencing air quality such as temperature, humidity, wing, rainfall and pressure.
Target	AQI	Air Quality Index (prediction Variable)

### Missing value imputation

**Fig. 2:**
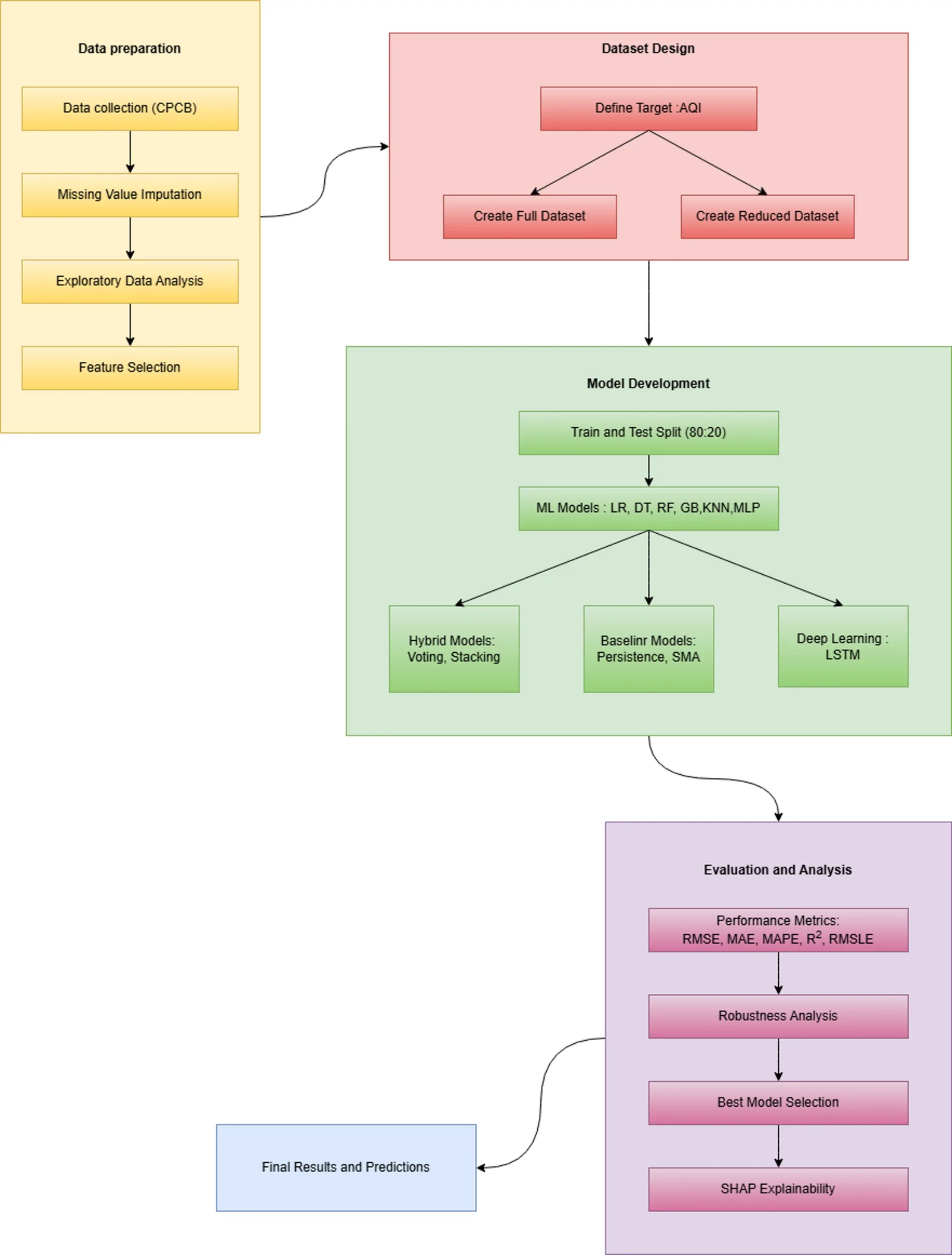
The overall architecture of AQI prediction.

The overall architecture of our proposed methodology is shown in Fig. 2. Missing data is a key aspect of time-series modelling to consider so that the data is complete and will not be biased in forecasting. To deal with the missing values and maintain the time-awareness of the dataset, a multi-step imputation strategy was used in the study. First, linear interpolation of short gaps (maximum of 3 consecutive hours) was time-based so as to provide smooth transitions between observations. Values of the same hour of the previous day (t -24) were then employed to capture daily periodic trends in medium gaps (up to 12 h). In cases of longer gaps (up to 24 h) the same hour of the preceding week (t − 168) was used to capture the weekly seasonality. Any missing values were imputed with hourly and monthly mean imputation, which does not destroy the trends of the data (diurnal and seasonal). Lastly, when there were still missing values, the global column mean was used as the last resort. This top-down methodology would make imputation be informed by temporal trends wherever feasible with minimum distortion of the underlying data distribution. Consequently, the processed dataset will not have any missing values and will be suitable to further machine learning and deep learning modelling. The complete algorithmic framework is provided in a separate supplementary file to facilitate better understanding of the proposed method.

### Feature selection

**Fig. 3:**
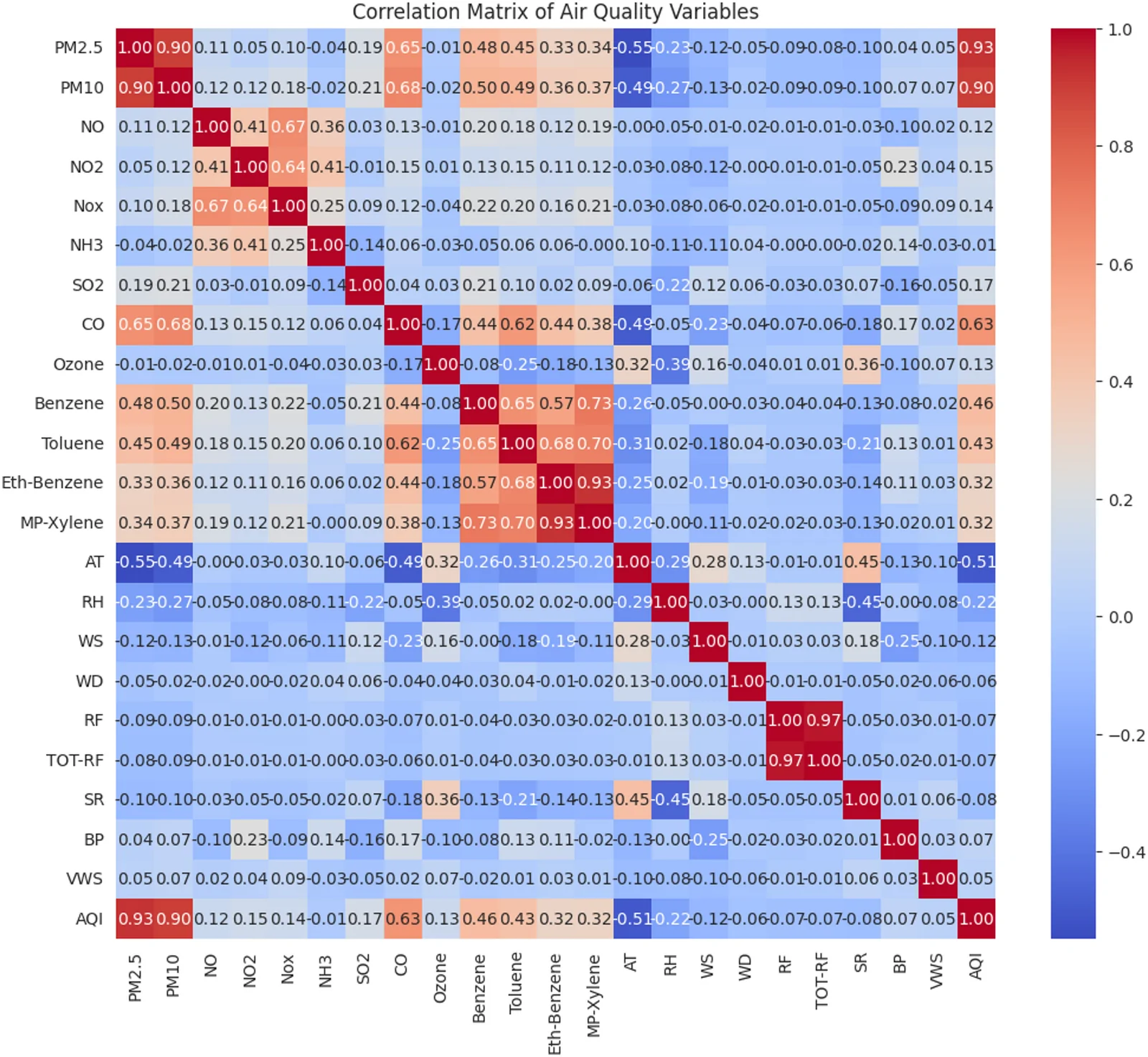
Correlation matrix for different variables.

The Fig. 3 illustrates the correlation matrix of various air quality and meteorological variables, highlighting the strength and direction of linear relationships among them. The Air Quality Index (AQI) shows a strong positive correlation with PM2.5 (0.93) and PM10 (0.90), confirming that these particulate pollutants are the main contributors to overall air quality degradation.

There is also moderate relationship between AQI and CO (0.63) and NOx (0.43) which demonstrates that vehicles and combustion emissions are major factors in air pollution. PM10 and PM2.5 among pollutants are almost perfectly correlated (0.90) with other sources. Likewise, benzene, toluene, and xylene display high inter-correlations, which imply common sources between vehicular and industries. Conversely, meteorological conditions like wind speed (WS), relative humidity (RH) and solar radiation (SR) bear a weak or negative correlation with the pollutants, which would mean that they disperse or dilute the air pollutants. In general, the matrix shows that gaseous and particulate contaminants are strongly connected, which create the most influential factors on the level of AQI in the research location.

**Fig. 4:**
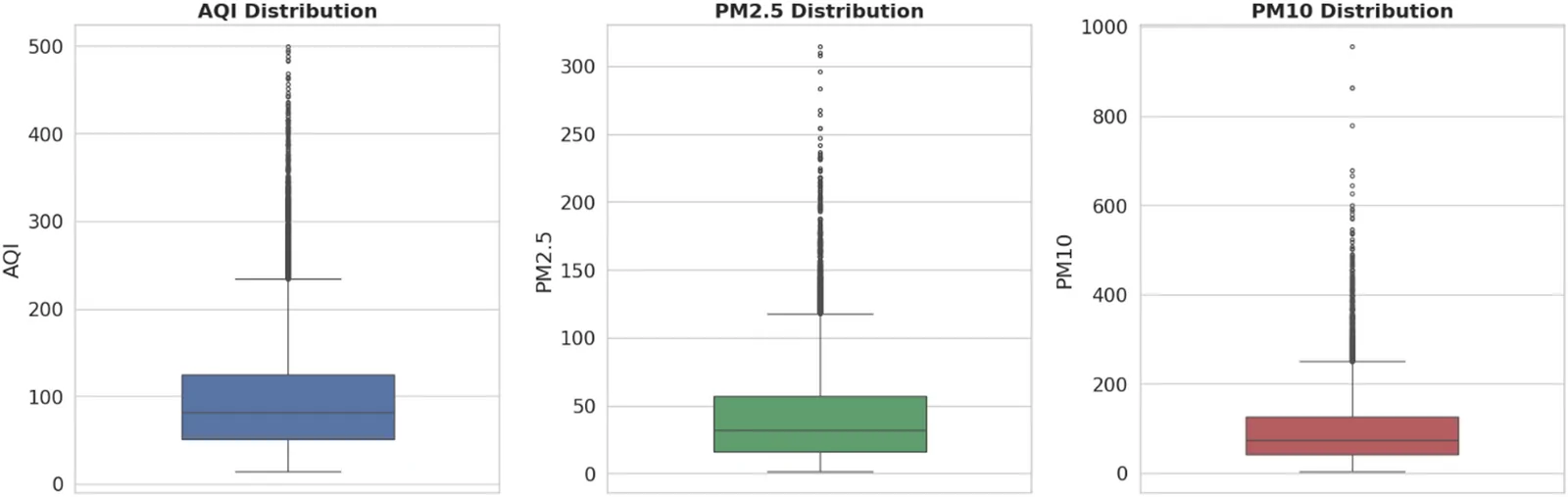
Box plots of AQI, PM2.5, PM10, and NO2,

The demonstration of dispersion and occurrence of outliers within each pollutant’s data range is shown in the box plot of AQI, PM2.5,PM10 and NO_2_ in Fig. 4. from the preceding histogram the median is situated nearer to the lower quartile which indicates a right skewed distribution.For each variable the inter quartile range (IQR) is quite narrow which indicates that majority of observations are within a modest range. Each plot displays a significant number of outliers above the top whisker, especially for AQI and PM10, which suggest sporadic high pollution levels. Many outliers were exhibited by PM2.5 and NO_2_ indicating transient surge in particle and nitrogen dioxide levels. the box plot shows that the air quality conditions is mild and the data always exhibit high value outliers, underscoring instance of severe pollution and fluctuations.

**Fig. 5:**
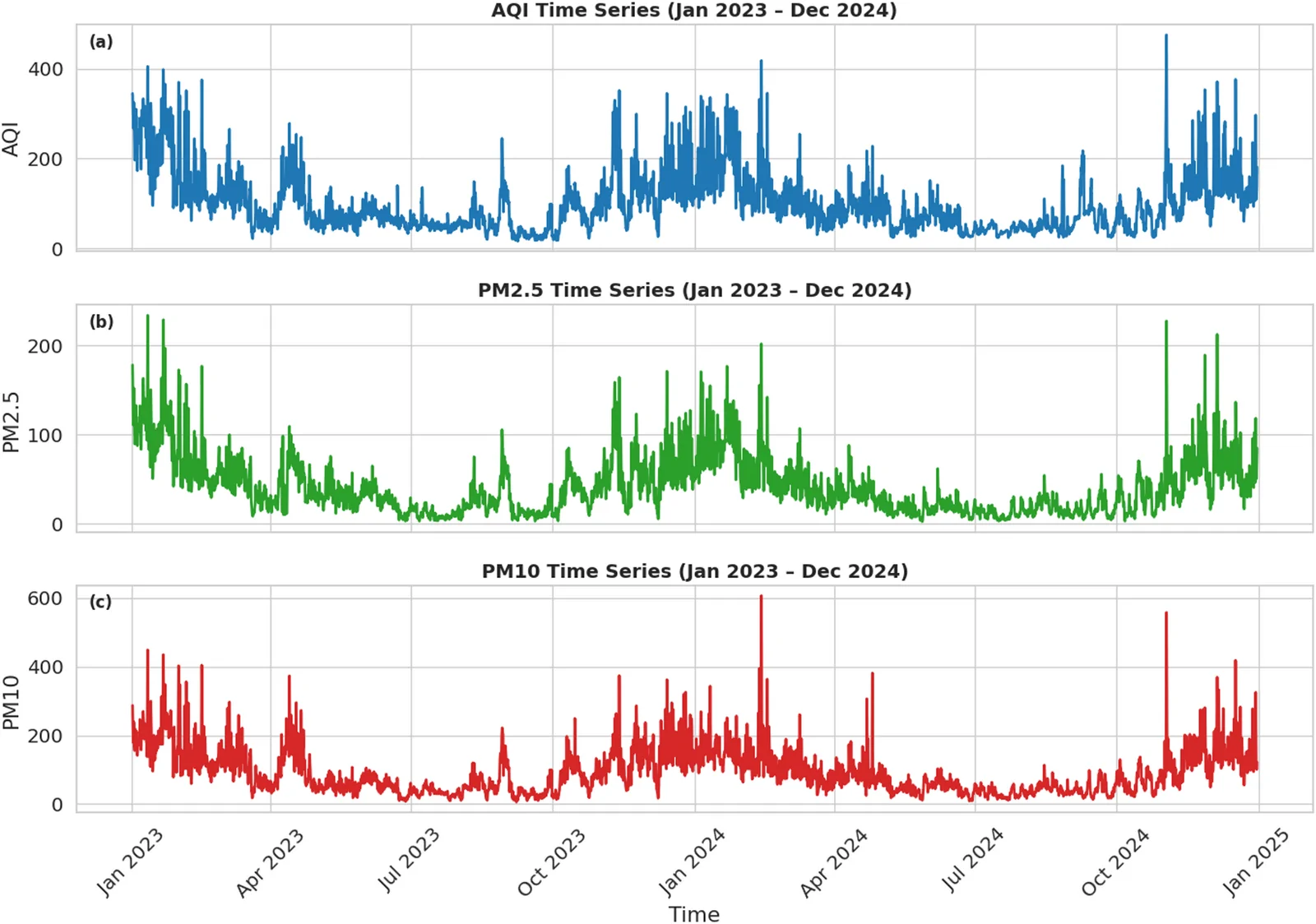
Time series plots for AQI, PM2.5 and PM10.

The Fig. 5. displays time series plots for AQI, PM2.5 and PM10 illustrating their fluctuations from January 2023 to April 2024. All four measures show significant temporal variations, indicating changes in air quality over various months and seasons.

The AQI, PM2.5, and PM10 graphs exhibit similar patterns, with peaks and troughs occurring concurrently, indicating that particulate matter significantly influences total air quality fluctuations. These peaks presumably align with winter months, during which reduced temperatures and stagnant air conditions confine contaminants to the surface. Conversely, reductions in pollution levels occur during the monsoon months, perhaps attributable to rainfall-induced purification of the atmosphere.

The NO₂ time series exhibits intermittent spikes, presumably associated with heightened vehicular or industrial activity; nevertheless, its fluctuations are less uniform than those of PM pollutants. The charts reveal distinct seasonal and temporal trends in air quality, underscoring the impact of climatic conditions and anthropogenic activities on pollution concentrations throughout time.

### Machine Learning methods

#### Multiple linear regression

Multiple linear regression (MLR) is a statistical modelling method which explains the relation between one dependent variable and two or more independent variables by assuming that they were related in a linear way. Here the dependent variable is represented as the linear combination of many predictors where each predictor is given a regression coefficient. The regression coefficient shows how it affects the response variable while other variables remains same. To model incorporates an intercept term and an error term in order to account for the fluctuations which cannot be explained by the random noise. Multiple linear regression is widely used in research and practical applications as it can get accurate prediction and show how the explanatory variables work together to affect the outcome.so it is highly helpful as a baseline and explanatory model in environmental pollution modelling.

1 $$\:y={\:\beta\:}_{0}+{\beta\:}_{1}{x}_{1}+{\beta\:}_{2}{x}_{2}+{\beta\:}_{3}{x}_{3}+\:\:\:\:.\:\:.\:\:.\:\:.\:\:.\:\:.\:\:.+{\beta\:}_{n}{x}_{n}+ϵ$$

Where:

- * y*= dependent variable (e.g., PM2.5 or PM10).
- $\:{x}_{1},{x}_{2},\dots\:,{x}_{k}$= independent variables (e.g., temperature, humidity, wind speed, traffic volume).
- $$\:{\beta\:}_{0}$$= intercept.
- $$\:{\beta\:}_{1},{\beta\:}_{2},\dots\:,{\beta\:}_{k}$$= regression coefficients.
- $$\:\epsilon\:$$ = error term.

### Decision tree

Supervisory machine learning models like decision trees organize data by feature values and predict outcomes. Overall, the goal of leaf node regression is to predict an average outcome. Internal nodes evaluate features for branches, and leaf nodes predict the result. Regression or classification models group similarly using MSE or Gini impurity/entropy. Decision trees are simple and show nonlinear relationships, but they may fit too well without chopping or regularizing^13^.

2 $${\text{Impurity reduction }} = \:I\left( P \right) - \frac{{\left| L \right|}}{{\left| P \right|}}I\left( L \right) - \frac{{\left| R \right|}}{{\left| P \right|}}I\left( R \right)$$

Where I(.) can be Gini index or Entropy.

Gini index: G = $$\:1-\sum\:_{k=1}^{K}{p}_{k}^{2}$$.

Entropy: H = $$\:-\sum\:_{k=1}^{K}{p}_{k}{\mathrm{log}}_{2}{p}_{k}$$.

### Random forest

Random Forest ensemble learning uses several decision trees for better accuracy and consistency. Regression averages tree outputs, and classification utilizes majority vote. Random data (bagging) and characteristics at each split train each forest tree. Reduces overfitting and improves tree generalization. Predictive modelling with non-linear correlations, high-dimensional data, outliers, and noise is possible using Random Forest. Combining three discoveries accomplishes this^14,20^.

3 $$\:\hat{y} = \frac{1}{T}\sum {\:_{{t = 1}}^{T} } f_{t} \left( x \right)$$

Where T is Number of decision trees, $\:{f}_{t}\left(x\right)$ is Prediction from the $\:{t}^{th}$ tree, $\:\widehat{y}$ is final predicted value (average of tree outputs).

### Gradient boosting

Gradient boosting builds a robust prediction model from weak learners like decision trees. New trees align with the loss function gradient to repair prior mistakes, “gradient boosting.” Each tree adds little to the forecast, lowering inaccuracy. Regularization and gradient boosting may optimize loss functions and decrease overfitting. Highly accurate yet expensive to process and susceptible to noisy data if uncalibrated.

4 $$\:r_{{im}} = - \left[ {\frac{{\partial \:L(y_{i} ,F\left( {x_{i} } \right))}}{{\partial \:F\left( {x_{i} } \right)}}} \right]_{{F = F_{{m - 1}} }}$$

Fit a weak learner $\:{h}_{m}\left(x\right)$ to residuals.

Update model $$\:{F}_{m}\left(x\right)={F}_{m-1}\left(x\right)+v.{h}_{m}\left(x\right)$$.

### K-Nearest neighbours

A non-parametric regression approach, KNN, averages a new data point’s k nearest neighbors’ Euclidean distance values. Although noisy data might be difficult, it works well for nonlinear connections. To avoid overfitting, Ridge Regression (L2 regularization) decreased coefficients without rejecting them by penalizing squared magnitude. This aids multicollinearity and numerous minors yet crucial predictors. Feature selection and model simplicity are simplified by Lasso Regression (L1 regularization) penalizing coefficient absolute values to zero. Lasso works best when few factors impact the goal^20^.

5 $${\text{For regression}}:\:\hat{y} = \frac{1}{K}\sum {\:_{{i = N_{k} \left( x \right)}} } y_{i}$$

For classification (majority voting):$\:\widehat{y}=\mathrm{arg}max\sum\:_{i={N}_{k}\left(x\right)}1({y}_{i}=c)$

Where $\:{N}_{k}\left(x\right)$ is the set of k nearest neighbours based on a distance metric (usually Euclidean distance).

### MLP regressor (Neural network)

MLP regressor is an artificial neural network-based regression model. It is used to capture the complicated and non-linear interactions between dependent variable and independent variables. Each layer is made up of neurons which are linked to each other and includes an input layer, one or more hidden layer and an output layer. Each neuron takes a weighted sum of its inputs, adds a bias term, and then runs the result via a nonlinear activation function like ReLU, sigmoid, or tanh. The model learns the best weights during training by employing backpropagation and gradient-based optimization techniques to minimize a loss function, which is usually the mean squared error. To teach the model the best weights by minimizing a loss function, backpropagation and gradient-based optimization methods were used which is usually the mean squared error.

6 $$y = f\left( {W_{2} g\left( {W_{1} x + b_{1} } \right) + b_{2} } \right)$$

were.

- x denotes the input feature vector,
- $\:{\mathbf{W}}_{1}$and $\:{\mathbf{W}}_{2}$represent the weight matrices of the hidden and output layers, respectively,
- $\:{\mathbf{b}}_{1}$and $\:{\mathbf{b}}_{2}$are the corresponding bias vectors,
- $\:g(\cdot\:)$is the nonlinear activation function applied in the hidden layer,
- $\:f(\cdot\:)$denotes the output activation function, and.
- * y*is the predicted output value (e.g., PM2.5 or PM10).

### Voting regressor

Voting regressor is an ensemble learning model which generates a single, final prediction by combining the results from several separate regression models. Each base model predicts the.

target value individually for the same input data. The results were subsequently aggregated by simple or weighted averaging. As a result, it often achieves greater generalization performance than any single regression model by itself.

7 $$\:\hat{y} = \frac{1}{M}\sum {\:_{{i = 1}}^{M} } f_{i} \left( x \right)$$

where:

- * M*is the number of base regressors,
- $\:{f}_{i}\left(\mathbf{x}\right)$denotes the prediction from the $\:{i}^{th}$regression model,
- **X** represents the input feature vector, and.
- $\:\widehat{y}$ is the final ensemble prediction.

To make things more stable, it combines predictions from Random Forest and Gradient Boosting using basic or weighted averaging.

8 $$\:\widehat{y}=\frac{{f}_{RF}\left(x\right)+{f}_{GBR}\left(x\right)}{2}$$

Where $\:{f}_{RF}\left(x\right)$ is predictions of random forest model and $\:{f}_{GBR}\left(x\right)$ is predictions of gradient boosting regressor.

### Stacking regressor

A stacking regressor is an advanced ensemble learning method tIat integrates the prediction accuracy by combining many basic regression models with a meta learner. In this method, several base regression models are first applied to the same set of data. These models include Random Forest, Gradient Boosting, and MLP. Then, their individual predictions are used as new input parameters for a second model, called a meta-regressor, which learns how to combine these predictions in the best way. In contrast to a vote regression that uses a simple average, stacking gives each model an amount of weight based on the data during the learning process. This lets it grasp complex relationships between the model results. For predicting the quality of the air, this method works really well because it uses the best parts of different models to give more accurate predictions of pollutants like PM2.5 and PM10.

9 $$\:\hat{y} = f_{{meta}} \left( Z \right) = f_{{meta}} \left( {f_{1} \left( x \right),f_{2} \left( x \right), \ldots \: \ldots \:.f_{M} \left( x \right)} \right)$$

Where:

- $\:{f}_{1},{f}_{2},\dots\:,{f}_{M}$are the base regressors (e.g., RF, GBR, MLP),
- $\:\mathbf{z}$is the vector of base model predictions,
- $\:{f}_{\mathrm{meta}}$is the meta-regressor that learns to optimally combine the base predictions,
- $\:\widehat{y}$ is the final predicted output (e.g., PM2.5 or PM10).

The system uses a meta-learner to find the best way to combine the predictions of several models, both linear and nonlinear.

10 $$\:\hat{y} = f_{{meta}} \left( {f_{{LR}} ,f_{{RF}} ,f_{{GBR}} } \right)$$

Where $\:{f}_{LR}\left(x\right)$ is predictions of linear regressor model, $\:{f}_{RF}\left(x\right)$ is predictions of random forest model and $\:{f}_{GBR}\left(x\right)$ is predictions of gradient boosting regressor.

### LSTM

Long Short-Term Memory (LSTM) is a type of recurrent neural networks (RNN) that is designed to learn temporal correlations within a sequence. Unlike traditional RNNs, LSTM solves the vanishing gradient problem through a gated architecture that incorporates input, forget and output gates. These gates regulate the flow of information and the model is able to retain the relevant past information over long periods of time.

11 $$\:y_{t} = f\left( {y_{{t - 1}} ,y_{{t - 2}} , \ldots \: \ldots \: \ldots \: \ldots \:y_{{t - n}} ,X_{t} } \right)$$

Where $\:{y}_{t}$ is the predicted AQI at time t, $\:{y}_{t-i}$ are past AQI values represents input features (pollutants and meteorological variables) and $\:f\left(\cdot\:\right)$ denotes the nonlinear mapping learned by the LSTM model.

#### Persistence model

Persistence model is a very basic time-series forecasting model that considers the future value to be the same as the last observed value. It is defined as:

12 $${\mathrm{AQI}}_{t} = {\mathrm{AQI}}_{{{\text{t - 1}}}}$$

Where $\:{AQI}_{t}$ is the predicted value at time t and $\:{AQI}_{t-1}$ is the AQI at the previous time step.

### Simple moving average

The SMA model predicts AQI as the average of the previous n observation.

13 $$\:AQI_{t} = \frac{1}{n}\sum {\:_{{i = 1}}^{n} } AQI_{{t - i}}$$

Where $\:{AQI}_{t}$ is the predicted value, n is the window size and $\:{AQI}_{t-i}$ represents past AQI values.

### Evaluation metrics

In this study, model performance was evaluated using standard regression metrics, namely the Coefficient of Determination (R²), Mean Absolute Error (MAE), Mean Squared Error (MSE), and Root Mean Squared Error (RMSE), to comprehensively assess prediction accuracy and error magnitude.

14 $$RMSE = \:\sqrt {\frac{1}{n}\:\sum {\:_{{t = 1}}^{n} } \left( {y_{t} - \overset{\lower0.5em\hbox{$\smash{\scriptscriptstyle\frown}$}}{y} _{t} } \right)^{2} }$$

15 $$MAE = \:\frac{1}{n}\sum {\:_{{t = 1}}^{n} } \left| {y_{t} - \overset{\lower0.5em\hbox{$\smash{\scriptscriptstyle\frown}$}}{y} _{t} } \right|$$

16 $$MAPE = \:\frac{1}{n}\sum {\:_{{t = 1}}^{n} } \left| {\frac{{y_{t} - \overset{\lower0.5em\hbox{$\smash{\scriptscriptstyle\frown}$}}{y} _{t} }}{{y_{t} }}} \right|{\mathrm{*}}100$$

17 $$R^{2} = 1\: - \frac{{\sum {\:_{{t = 1}}^{n} } \left( {y_{t} - \overset{\lower0.5em\hbox{$\smash{\scriptscriptstyle\frown}$}}{y} _{t} } \right)^{2} }}{{\sum {\:_{{t = 1}}^{n} } \left( {y_{t} - \bar{y}_{t} } \right)^{2} }}$$

18 $$RMSLE = \:\sqrt {\frac{1}{n}\sum {\:_{{t = 1}}^{n} } \left( {{\mathrm{log}}_{e} \left( {\overset{\lower0.5em\hbox{$\smash{\scriptscriptstyle\frown}$}}{y} _{t} + 1} \right) - {\mathrm{log}}_{e} \left( {y_{t} + 1} \right)} \right)^{2} }$$

Where $\:{y}_{t}$ is actual value and $\:\widehat{{y}_{t}}$ is the predicted value respectively.

## Results and discussion

**Table 2:** Performance comparison of machine learning, ensemble, baseline, and deep learning models using the full feature set for AQI prediction.

Models	Features	RSME	MAP	MAPE	RMSLE	*R* ^2^
Linear Regression	Full	25.374	15.979	17.028	0.223	0.860
KNN	Full	40.903	26.089	24.957	0.463	0.635
MLP	Full	17.796	9.485	9.382	0.132	0.931
Decision Tree	Full	13.805	2.705	2.274	0.116	0.958
Random Forest	Full	12.028	2.616	2.165	0.083	0.968
Gradient Boosting	Full	11.018	4.811	5.306	0.092	0.974
Voting Regressor	Full	10.938	3.526	3.580	0.079	0.974
Stacking Regressor	Full	11.193	3.231	3.328	0.078	0.973
Persistence (Baseline)	N/A	18.697	8.972	9.017	0.150	0.924
SMA (Baseline)	N/A	26.257	14.024	13.433	0.194	0.850
LSTM	Full	20.247	12.665	13.536	0.275	0.911

From Table 2. We can observe that Ensemble and tree-based models perform best in predicting AQI when the entire feature set is used. The Voting Regressor has the lowest RMSE (10.938) and high R^2^ of 0.974 whereas the Stacking Regressor has the lowest MAE and MAPE, which means it is more accurate. Gradient Boosting and Random Forest are also doing good, followed by Decision Tree. On the contrary, Linear Regression and KNN demonstrate lower accuracies which indicates that the methods have shortcomings in their ability to model non-linear relationships. The MLP model works average. Baseline models are less precise and capture the trends of time, but it does not outperform the ensemble approaches better than LSTM. On the whole, hybrid ensemble models are the most trusted in terms of predicting AQI.

**Table 3:** Performance comparison of machine learning and ensemble models using the reduced feature set (excluding PM2.5 and PM10) for AQI prediction.

Models	Features	RSME	MAP	MAPE	RMSLE	*R* ^2^
Linear Regression	Reduced	47.070	30.485	30.659	0.385	0.517
KNN	Reduced	55.777	37.010	33.959	0.575	0.322
MLP	Reduced	56.325	40.972	41.718	0.764	0.309
Decision Tree	Reduced	52.988	34.170	33.903	0.456	0.388
Random Forest	Reduced	41.251	26.070	25.227	0.341	0.629
Gradient Boosting	Reduced	41.861	26.239	25.121	0.358	0.618
Voting Regressor	Reduced	40.773	25.527	24.469	0.338	0.638
Stacking Regressor	Reduced	42.052	27.908	27.694	0.421	0.615

As depicted in Table 3. Models that are trained on the smaller feature set exhibit a strong drop in performance on all measures. Voting Regressor has the highest results among them (R^2^ = 0.638, RMSE = 40.773) whereas the other two are Random Forest and Gradient Boosting, which however have higher errors in comparison with full-feature models. Linear Regression, Decision Tree, KNN, and MLP cannot work well, and the values of R^2^ decrease to 0.309. In general, the trend of RMSE and MAPE growth of all the models demonstrates the loss of predictive power of the models in the absence of the key particulate variables, which proves that they are essential in predicting AQI.

**Fig. 6:**
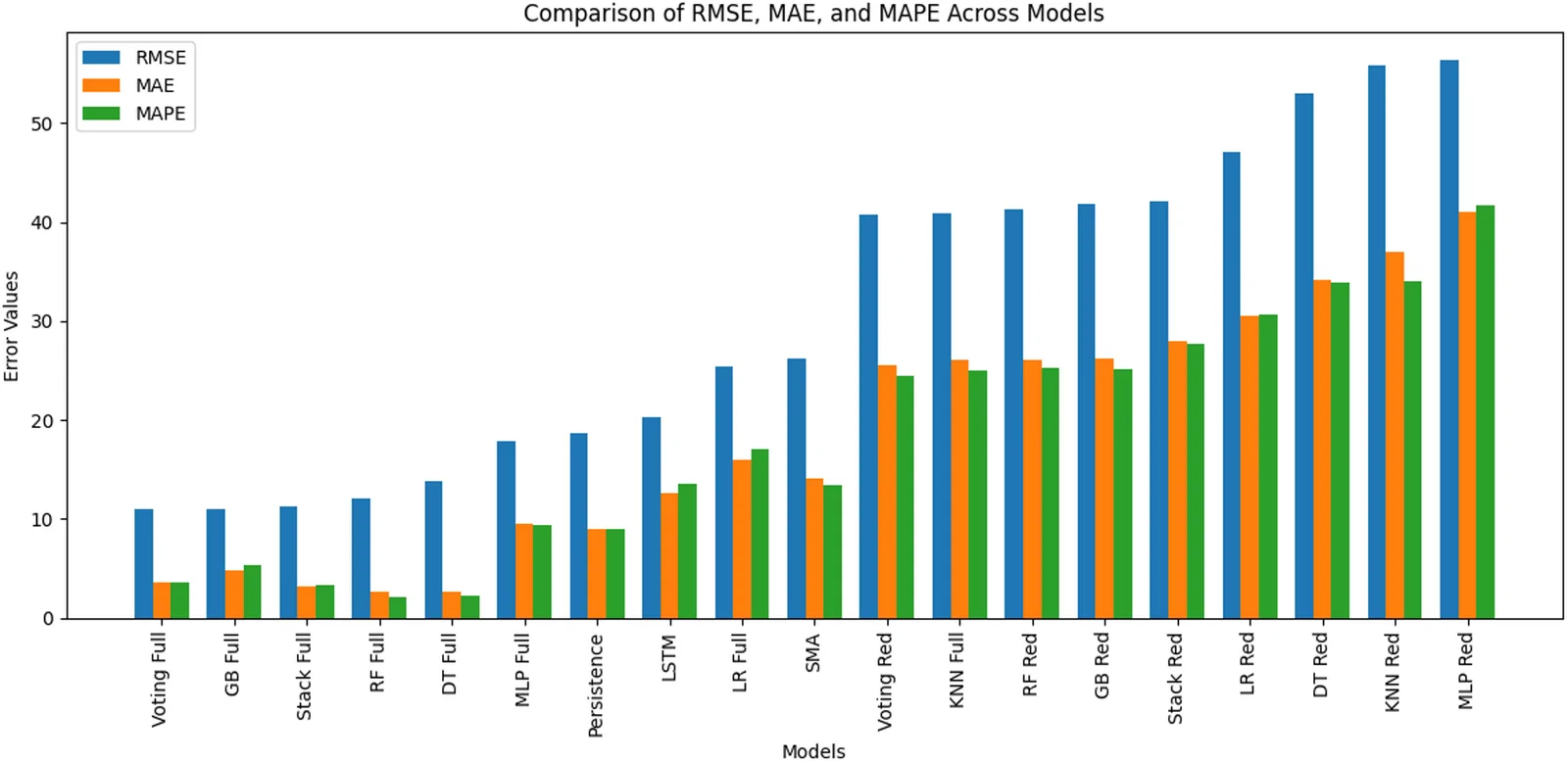
Comparison of RMSE, MAE, and MAPE for Hybrid/Ensemble Models.

Figure 6. shows a relative comparison of the error-based performance measures, such as RMSE, MAE, and MAPE, of all models in their full and reduced feature versions. The findings make it clear that models that are trained using the entire set of features have much lower error values than models that are reduced. The Voting Regressor (Full) has the lowest RMSE and the Stacking Regressor (Full) has the lowest MAE and MAPE, which confirms the better predictive power of ensemble methods. The tree-based models like Gradient Boosting, Random Forest and Decision Tree are also performing well with relatively low error values, but Linear Regression and KNN have relatively high errors, indicating their low ability to capture complex and non-linear relationships. Conversely, every reduced-feature model shows a significant growth in error measures, and the RMSE values are above 40 and the MAPE values are over 25% in most instances. MLP and KNN are especially sensitive to the loss of important variables like particulate matter, which makes them especially vulnerable to the process of degradation. The baseline models such as Persistence and SMA have moderate levels of error, meaning that temporal patterns do exist, but are not enough to predict accurately without more information about the features. The LSTM model is superior to the baseline methods but not to the ensemble methods. All in all, the figure supports the fact that the hybrid ensemble models that have been trained on a complete set of features are the most accurate and reliable in terms of prediction of AQI.

**Fig. 7:**
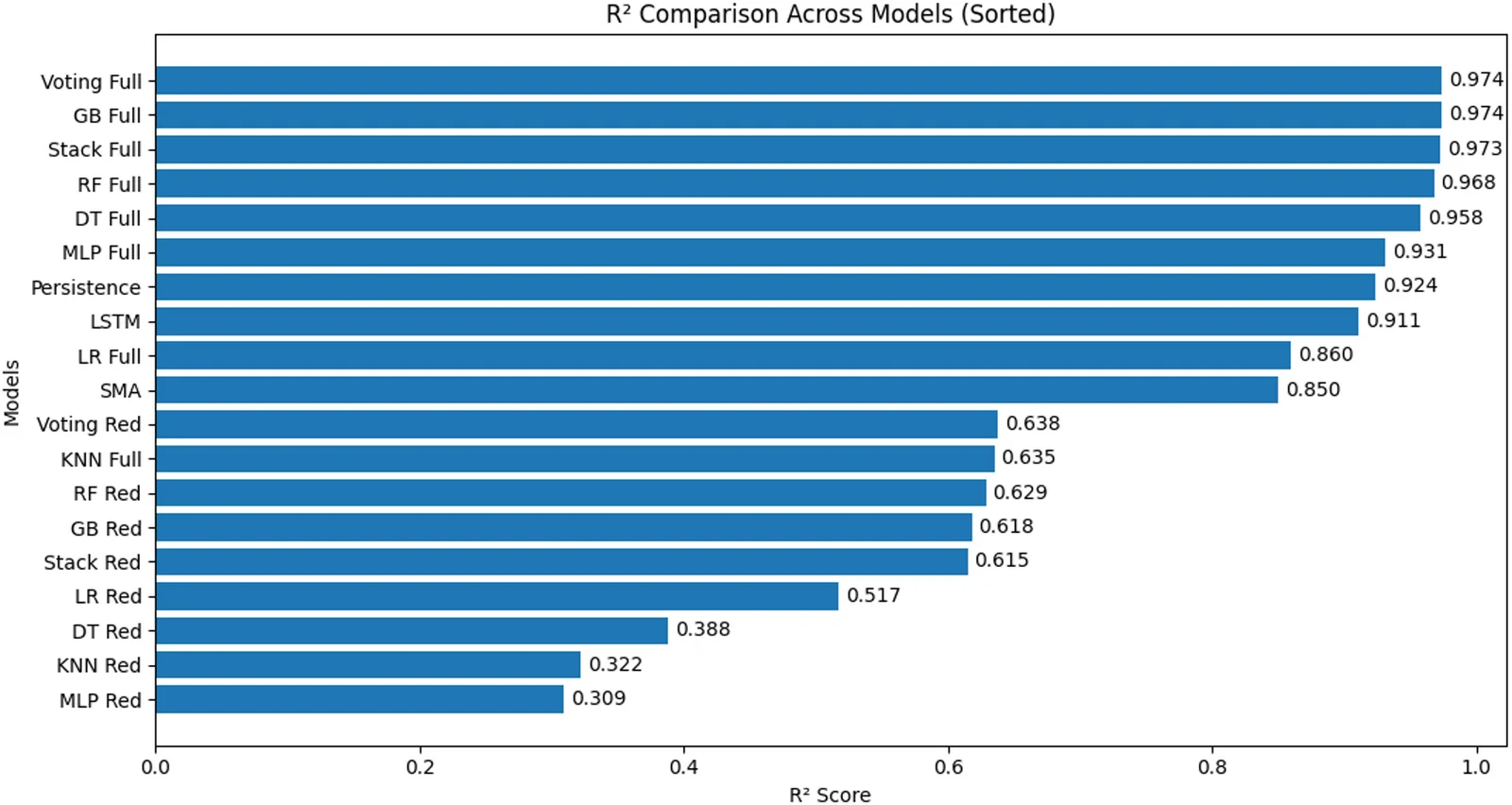
R^2^ Comparison of hybrid regression models.

Figure 7. compares the R^2^ of all models, with higher values representing a better predictor. The results are clear indicating that the full-feature ensemble models are the best in terms of accuracy with the Voting Regressor and Gradient Boosting (﻿R^2^ = 0.974) having the highest accuracy followed by the Stacking Regressor and the Random Forest. This proves the applicability of ensemble learning in representing the complex relationships in AQI data. The LSTM (R^2^=0.911) model is reasonably good, but not better than the ensemble methods. Moderate performance is observed in baseline models like Persistence (0.924) and SMA (0.850). Contrary to that, the models that are trained on reduced features demonstrate a steep fall in the values of R^2^, with the worst results of MLP, KNN, and Decision Tree, which indicates that the variables of particulate matter (PM2.5 and PM10) are key contributors to accurate prediction of AQI.

**Fig. 8:**
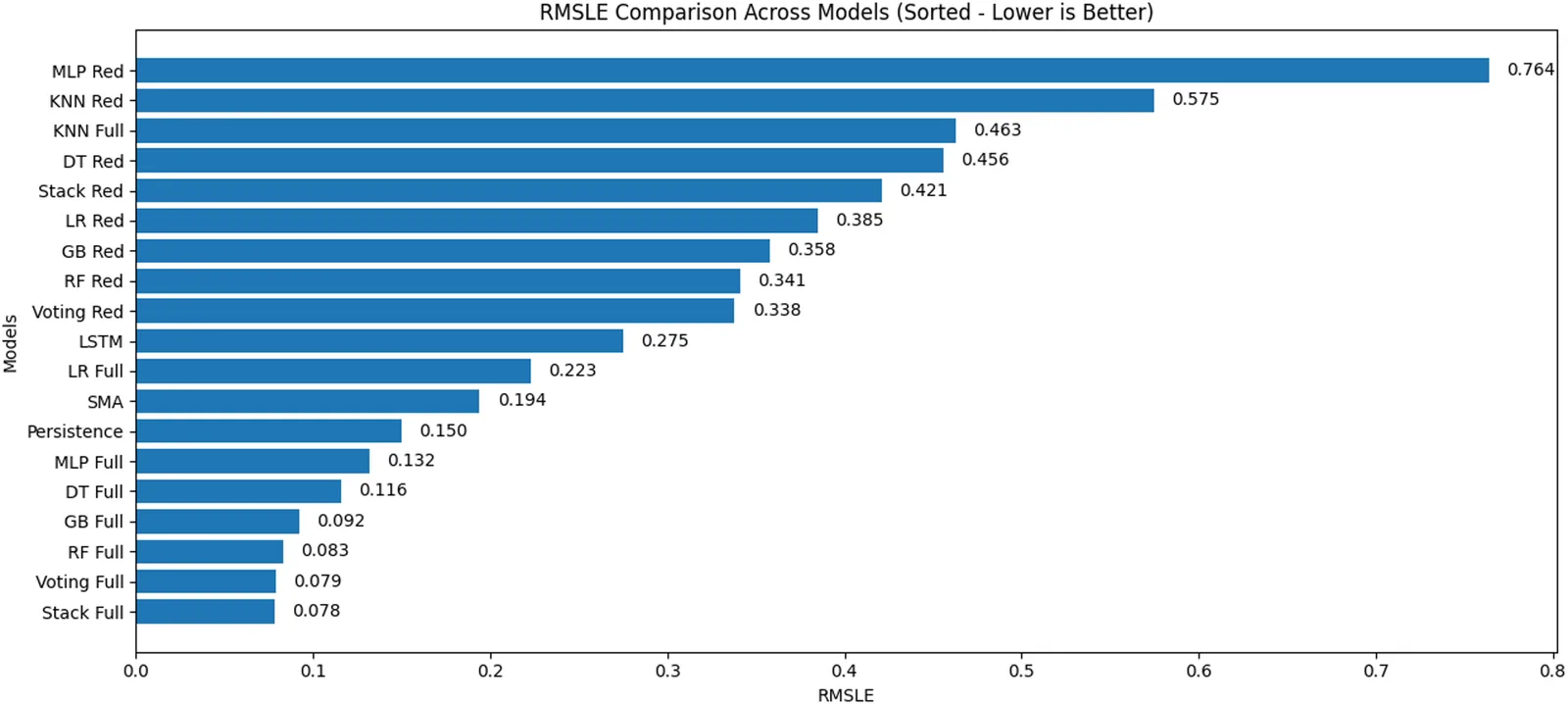
RMSLE comparison of hybrid regression models.

Figure 8. presents the comparison of RMSLE of all models, a lower value is better performance. It is clear that ensemble models with full feature, especially Stacking Regressor (0.078) and Voting Regressor (0.079) have the lowest error, and then there is the Random Forest and Gradient Boosting. This underscores the high ability of ensemble methods to deal with variability and minimise the errors of prediction. Conversely, models that have been trained on reduced features depict a considerably high RMSLE value where MLP and KNN have the worst performance, thus suggesting that important information is being lost during the omission of PM2.5 and PM10. The baseline models like the Persistence (0.150) and SMA (0.194) are an average of accuracy but still demonstrably lower than the machine learning models. The LSTM model (0.275) is a good performer but not better than the ensemble models implying that ensemble learning is more efficient than the standalone deep learning models using structured tabular AQI data.

**Fig. 9:**
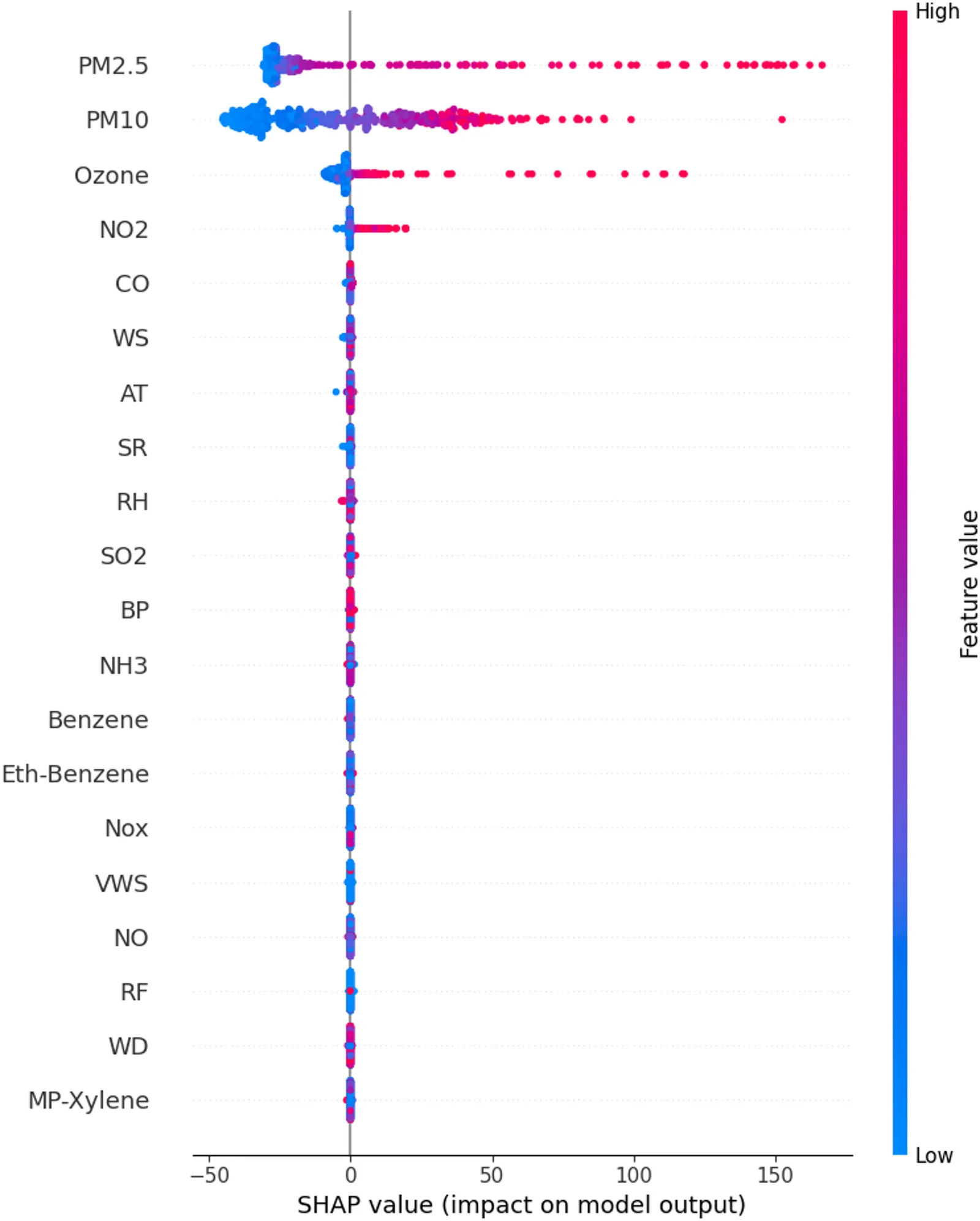
SHAP Value impact on model output.

Figure 9. shows SHAP summary plot of the most effective model (Voting Regressor), which shows how each feature contributes to predictions of AQI. It is evident that PM2.5 and PM10 are the most significant variables, with the most dispersed SHAPs values and maximum effects on the model output. These pollutants are strongly correlated with positive SHAP values (Higher concentrations of these pollutants (indicated by red points) and lower concentrations (blue points)) resulting in higher AQI levels and lower AQI predictions respectively. Ozone (O_3_) and NO_2_ are moderate in their contribution with observable variation in their impact implying their contribution to the formation of secondary pollution and their interaction in the atmosphere. Conversely, the meteorological variables wind speed (WS), temperature (AT), relative humidity (RH) and solar radiation (SR) have relatively lower SHAP magnitude, suggesting secondary but significant contribution to air quality modulation. The rest of the variables, such as volatile organic compounds (e.g., benzene, ethyl-benzene, and xylene) have little effect, and the SHAP values are concentrated around 0, indicating that they do not play a significant predictive role in the data. Altogether, the SHAP analysis indicates that particulate matter is the primary predictor of AQI that other pollutants and weather conditions give corroborative data. It should be mentioned that these results represent the importance of features and associations in the model and not should be considered as a cause and effect relationship.

### Limitations and feature scope

This study is subject to certain limitations. The sample is also very narrow to one city and time, which can impact the applicability of the findings to other areas. Even though it incorporated the main pollutant and meteorological variables, other factors like traffic, industrial emissions as well as satellite data were not factored in. As AQI is based on the concentration of pollutants, the inclusion of such variables as PM2.5 and PM10 can create some inherent dependency, however, it was countered with reduced-feature analysis. In modeling terms, the LSTM implementation is based on a simplified temporal structure and this might restrict its capacity to capture long term dependencies. The existing feature coverage is based on fundamental environmental variables, and wider spatial and social-economic variables have not been examined. These should be included in future work to enhance the generalization of the model and its applicability in the real world.

## Conclusion

This paper outlines an extensive model of predicting the Air Quality Index (AQI) based on machine learning, hybrid ensemble models, and deep learning models. To evaluate the overall performance of models, deal with possible target leakage, and determine the impact of key pollutants, a more detailed experimental analysis was carried out with full and reduced feature configurations. The findings of the studies all indicate that models that are trained with the complete set of features are much better at predicting AQI than those that are trained with reduced features, and that particulate matter, especially PM2.5 and PM10, are very important in AQI prediction. Out of all the considered models, ensemble methods, i.e. the Voting and Stacking regressors, performed the best in general. The Voting Regressor had the lowest RMSE(10.938) and highest R^2^ (0.974) and Stacking Regressor had the lowest MAP (3.231) and MAPE (3.328) which showed better predictive accuracy and strength. Random Forest and Gradient Boosting (individual models) were also effective but simpler models, including Linear Regression and KNN, demonstrated relatively limited ability to capture a complex, non-linear relationship. Simple Moving Average and Persistence showed the existence of the temporal dependence in the data but could not be used to provide high-accuracy predictions. The LSTM model showed some average performance, being better than the baseline methods but not as good as ensemble models. The robustness analysis further confirmed the stability and generalization ability of the proposed framework with stable performances through various temporal splits. Also, SHAP-based explainability helped obtain some insightful information on feature importance, which validates the hypothesis that particulate matter is the main driver of AQI prediction, and meteorological variables and other pollutants are of secondary importance. Notably, these understandings are not causal, but predictive. In general, the hybrid machine learning framework that has been proposed has a high degree of accuracy, robustness, and interpretability and thus can be used as a reliable tool in AQI prediction and environmental monitoring. The results of this research can be used to inform policymakers and environmental agencies in making informed decisions on air quality management. Future directions are to add more spatial and temporal features, consider more advanced deep learning architectures, and generalize the framework to multi-city or real-time forecasting tasks.

### Novelty of the study

The study’s innovation is the creation of a hybrid machine learning frame work for AQI prediction that incorporates individual, ensemble and deep learning models in to a single evaluation frame work. In contrast to the traditional methods, this work intentionally excludes PM2.5 and PM10 in order to assess the impact of the target reliance by comparing full and reduced feature configurations.

The study also presents a new validation method that takes leakage factors in to consideration by employing robustness analysis across various periods of time and chronological splitting. The implementation of baseline time-series models like persistence and SMA provides a realistic standard for performance evaluation. SHAP based explainability also improves interpretability by identifying significant aspects. The combined use of hybrid modelling, leakage analysis, robustness validation and explainability establishes a comprehensive and reliable frame work for AQI forecasting.

## Supplementary Information

Below is the link to the electronic supplementary material.

Supplementary Material 1

## Data Availability

Upon a reasonable request, the sources and other pertinent information will be provided.
