# Supplementary material for: Regression based hybrid machine learning model performance evaluation on air quality index prediction in Kolkata
Source: Sci Rep. 2026 Jun 10;16:26399. doi: 10.1038/s41598-026-54918-x (PMC13500472; doi:10.1038/s41598-026-54918-x)
Supplement: Supplementary file 1 [file 41598_2026_54918_MOESM1_ESM.docx]

**Supplementary file for Algorithm**

**Algorithm 1:**

**Proposed Hybrid Machine Learning Framework for AQI Prediction**

**Input:**

Air quality dataset D consists of the feature matrix X with pollutant and metrological variables and y(AQI) as the target variable.
**Output:**

Anticipated AQI values in addition to model evaluation metrics.

**Step 1: Data Acquisition**

Download the Central Pollution Control Board’s (CPCB) air quality data set, to make sure time stamped measurements of pollutant concentrations and metrological variables.

**Step 2: Data Preprocessing and Missing Value Imputation**

Handle missing values by using hierarchical muti-step imputation technique to clean and process the dataset.

- For short gaps (≤ 3 hours) use linear interpolation.
- For medium and long gaps (previous day $t-24$, previous week $t-168$) use lag-based imputation.
- Use hourly/monthly mean and global mean to fill the rest of the values.

**Step 3: Exploratory Data Analysis (EDA)**

To learn more about data use statistical analysis

- Examine the patterns throughout time and observe how features are distributed.
- Figure out the relation between variables by computing correlation matrix.

**Step 4: Feature Selection**

Basing on correlation analysis and domain experience identify essential features, to identify significant pollutant and metrological variables.

**Step 5: Dataset Preparation**

Define:

- Target variable: $y=\text{AQI}$
- Feature matrix: $X$

Construct two experimental datasets:

- Full Feature Set: All variables included
- Reduced Feature Set: Excluding PM2.5 and PM10 to mitigate potential target dependency

**Step 6: Train–Test Splitting**

Split the dataset chronologically to preserve temporal order:

- Training set: First 80% of observations
- Testing set: Remaining 20%

**Step 7: Individual Model Training**

For both feature sets train several machine learning models.

- Linear Regression
- Decision Tree Regressor
- Random Forest Regressor
- Gradient Boosting Regressor
- K-Nearest Neighbors (KNN)
- Multi-Layer Perceptron (MLP)

**Step 8: Hybrid Ensemble Model Construction**

To improve prediction accuracy, develop ensemble machine learning models.

- Voting Regressor: Combine Random Forest and Gradient Boosting predictions
- Stacking Regressor:
  - Base learners: Random Forest, Gradient Boosting
  - Meta-learner: Linear Regression
  - Use k-fold cross-validation to generate out-of-fold predictions and avoid leakage

**Step 9: Baseline Time-Series Models**

For comparison baseline approaches should be implemented.

- Persistence Model: $AQI_{t}=AQI_{t-1}$
- Simple Moving Average (SMA): Mean of previous $n$ observations

**Step 10: Deep Learning Model**

To capture temporal dependencies in the data train a Long Short-Term Memory (LSTM) network.

**Step 11: Model Evaluation**

Standard performance metrics should be used to assess all models.

- Root Mean Square Error (RMSE)
- Mean Absolute Error (MAE)
- Mean Absolute Percentage Error (MAPE)
- Root Mean Square Logarithmic Error (RMSLE)
- Coefficient of Determination ($R^{2}$)

**Step 12: Robustness Analysis:**

To check the stability of the model use periodic time-aware validation.

- Carry out experiments using different durations. (Windows that roll and expand)
- Evaluate mean and standard deviation of performance metrics.

**Step 13: Model Selection**

Basing on overall performance select the best model which gives lowest RMSE and highest R^2^.

**Step 14: Model Explainability**

To understand things easier apply SHAP (Shapley Additive exPlanations)

- Employ TreeExplainer on the optimal model (Voting Regressor)
- Analyse feature significance and its impact on predictions.

**Step 15: Output Generation**

Report:

- Final predicted AQI values
- Comparative model performance metrics
- SHAP-based feature importance analysis

**Algorithm 2:**

**Proposed Voting Regressor Framework for AQI Prediction**

**Input:**
Training dataset $\left( X_{train} , y_{train} \right)$, Testing dataset $\left( X_{test} \right)$

**Output:**
Predicted AQI values $\hat{y}$and performance metrics

**Step 1: Initialize Base Models**

- Define Random Forest Regressor (RF)
- Define Gradient Boosting Regressor (GBR)

**Step 2: Construct Voting Regressor**

- Combine base models into a Voting Regressor:

$$\hat{y}=\frac{1}{n}\sum_{i=1}^{n} f_{i}(X)$$

where $f_{i}$represents predictions from each base model (RF and GBR)

**Step 3: Model Training**

- Fit the Voting Regressor on the training data:

$$\text{Model.fit}(X_{train},y_{train})$$

**Step 4: Prediction**

- Generate predictions on test data:

$$\hat{y}=\text{Model.predict}(X_{test})$$

**Step 5: Evaluation**

- Compute performance metrics: RMSE, MAE, MAPE, RMSLE, and $R^{2}$

**Step 6: Output Results**
